# Identification of diagnostic genes in rheumatoid arthritis using integrated bioinformatics, machine learning, and experimental validation

**DOI:** 10.3389/fmed.2026.1803764

**Published:** 2026-04-13

**Authors:** Zhibin Zhang, Huimin Fang, Jianen Guo, Yutang Zhao, Zhihua Zhan, Jiri Mutu Zhang

**Affiliations:** 1 College of Traditional Chinese Medicine, Chengde Medical University, Chengde, Hebei, China; 2 NMPA Key Laboratory for Quality Control of Traditional Chinese Medicine (Mongolian Medicine), Mongolian Medical College, Inner Mongolia Minzu University, Tongliao, Inner Mongolia, China

**Keywords:** bioinformatics, diagnostic genes, differentially expressed genes, immune infiltration, rheumatoid arthritis

## Abstract

**Background:**

Rheumatoid arthritis (RA) and osteoarthritis (OA) are prevalent joint diseases with overlapping clinical manifestations but distinct pathogenesis and treatment strategies. Misclassification may lead to inappropriate management. Therefore, accurate molecular discrimination between RA and OA is important. This study aimed to identify diagnostic genes associated with RA, with a particular emphasis on distinguishing RA from OA using integrated bioinformatics and machine learning approaches.

**Methods:**

Public GEO transcriptomic datasets were analyzed to identify differentially expressed genes (DEGs) between the RA group and comparison groups. LASSO and SVM-RFE algorithms were applied for feature selection. Immune cell infiltration was estimated using the ssGSEA algorithm. A protein–protein interaction (PPI) network and transcription factor analysis were performed to explore potential regulatory mechanisms. Drug sensitivity analysis based on CellMiner IC50 data was conducted as an exploratory approach. *In vitro* validation was performed using TNF-α–stimulated HFLS-RA cells, followed by RT–qPCR analysis.

**Results:**

Three key genes—EPYC, MAGED1, and LAP3—were identified as overlapping features between the LASSO and SVM-RFE models. ROC analysis demonstrated good discriminatory performance (AUC > 0.85). EPYC and LAP3 were associated with immune cell infiltration patterns. TNF-α stimulation significantly modulated the mRNA expression of these genes in the HFLS-RA cells.

**Conclusion:**

EPYC, MAGED1, and LAP3 are inflammation-associated genes with potential diagnostic relevance in RA. Further validation in larger independent cohorts and protein-level studies is needed to confirm their clinical applicability.

## 1 Background

Rheumatoid arthritis (RA) represents a chronic and progressive autoimmune inflammatory disease which can affect approximately 1% of the global population (1). Progressive synovial inflammation gradually erodes the affected joint tissue, resulting in irreversible damage. RA typically presents as joint stiffness, swelling, and pain, and imposes a substantial burden on individuals and society (2). Developing methods for early screening of RA will reduce its impact on patients and society. Currently, rheumatoid factor (RF) and anti-cyclic citrullinated peptide (anti-CCP) antibodies are applied as serum markers for diagnosing and classifying RA in clinical practice (3). Antibodies against peptidyl arginine deiminase-4 (PAD-4) (4) and glucose-6-phosphate isomerase (anti-GPI) (5) have also been indicated to be diagnostic or prognostic biomarkers for RA. However, because RF and anti-CCP autoantibodies have not been verified in a prospective cohort and their clinical relevance is unclear, these markers have not been widely used. Therefore, it is critical to explore new and effective autoantibodies for early prevention and treatment of RA.

Transcriptome and microarray analysis technology has been successfully used to find new diagnostic markers in various tumors and RA (6–8). Bioinformatics analysis methods that integrate disease-related variations with different genomic and other high-throughput data provide new clinical insights and treatment options for RA. This research strategy can be used to analyze the transcriptomic profile in RA synovial tissues, thus facilitating the identification of novel potential biomarkers that could help improve the efficiency of RA diagnostics and treatment. Studies have already identified MTFI and RUNX1 as potential new therapeutic targets for RA (9, 10). In addition, bioinformatics analyses have revealed that new autoantibodies, such as anti-PTX3 and anti-DUSP11, have potential value as biomarkers for diagnosing anti-CCP–negative RA patients (11).

In this study, R software was adopted for downloading the RA expression profile dataset from the GEO database; the chip data were then corrected between groups using the limma (12) package and displayed using a PCA map. DEGs were calculated using the limma method within the limma package. Using the clusterProfiler (13) package, Gene ontology (GO) enrichment and Kyoto Encyclopedia of Genes and Genomes (KEGG) pathway analyses were conducted on the DEGs. Gene set enrichment analysis (GSEA) was carried out by adopting the clusterProfiler package, and immune infiltration levels were calculated on the basis of the ssGSEA algorithm (R software GSVA package). Then, a differential analysis of the infiltration level of immune cells was performed. In addition, we also assessed intersecting autophagy phenotype-related genes among the DEGs using GeneCards and performed an enrichment analysis. The pheatmap package was applied to draw the thermogram of the immune infiltration level of immune cells in osteoarthritis and RA. With the purpose of improving the performance of the diagnostic model, we screened feature variables based on the machine learning method of the support vector machine in order to find the best variables and more accurate biomarkers for the diagnosis and discrimination of osteoarthritis and RA. In addition, Protein–Protein Interaction (PPI) networks were constructed to further investigate the different biological mechanisms of RA compared with osteoarthritis. The regulatory relationship between genes was visualized using Cytoscape software, and the network’s topological properties were calculated for analysis. We identified miRNA target gene interaction pairs and significant crossover DEGs, mapped miRNA–mRNA (target gene) networks, and analyzed transcription factors that potentially regulate signature genes. Finally, a correlation analysis was conducted between characteristic genes and immune cell infiltration levels, and a drug sensitivity analysis was performed to evaluate the therapeutic potential of several FDA-approved, clinically tested drug molecules targeting the screened genes. A correlation map was generated to visualize the gene expression and IC50 data for significantly related compounds. In the present study, bioinformatics analysis, machine learning, and experimental validation were combined to identify potential diagnostic biomarkers for rheumatoid arthritis. By directly comparing RA with osteoarthritis, we further aimed to improve the specificity of biomarker screening and to identify molecular features more closely associated with RA than with common inflammatory changes shared by joint diseases. These findings may provide additional insight into the differential diagnosis of RA and support future studies on more precise diagnostic strategies.

## 2 Materials and methods

### 2.1 Data download and preprocessing

The GEOquery package of R software (version 4.0.3)^1^ was employed to download RA expression profile datasets with a reliable sample source from the GEO^2^ database. The samples in the datasets were obtained from *Homo sapiens*. The mRNA data were obtained from the GSE55235 (14) and GSE153015 (15) datasets. The GSE55235 dataset was obtained from the GPL96 platform and included 10 control normal human joint samples, 10 osteoarthritic joint samples, and 10 RA tissue samples. The GSE153015 dataset was obtained from the GPL570 platform and included 4 osteoarthritis joint tissue samples and 20 RA tissue samples. The miRNA data were obtained from the GPL20870 platform derived from the GSE72564 dataset and included 4 osteoarthritis joint tissue samples and 4 RA tissue samples.

All datasets were read using the GEOquery package (16) (see text footnote 2). In addition, the probe name annotations were used for the chip GPL platform files. All raw expression matrices were log2-transformed and normalized using the normalizeBetweenArrays function in the limma package. Batch effects were identified by principal component analysis (PCA) and corrected using the removeBatchEffect function, where the batch parameter was set according to platform information and the design matrix included the group variable. All analyses were performed in R 4.0.3 with a fixed random seed of 123 to ensure reproducibility. The impact of calibration between samples is shown in PCA constructed with the FactoMineR package.

### 2.2 Screening and functional analysis of DEGs

DEGs were identified in the control versus osteoarthritis group and the control versus RA group in the GSE55235 dataset using the limma method of the limma package (significant differences were required to satisfy adj. *p* value < 0.05 and |log2FC| > 1), and volcano plots of the DEGs were generated with the use of the ggplot2 package.

GO (17) enrichment and KEGG pathway analyses were carried out based on the clusterProfiler package for the DEGs in the osteoarthritis and RA groups, respectively, and histograms and functional clustering networks were plotted using the enrichplot Package (18). The species was limited to *Homo sapiens*. In addition, an adjusted *p* value (adjustment method: Benjamini–Hochberg method) less than 0.05 indicated statistical significance.

GSEA (19) was also performed using the clusterProfiler package for the osteoarthritis and RA groups separately to analyze the activated or suppressed role of the disease group relative to the control group in the classical pathway. The “c2.all.v7.4.symbols.gmt” was determined to be the reference gene set. False discovery rates (FDR) < 0.25 and *p* values < 0.05 indicated significant enrichment.

### 2.3 Calculation of the level of immune cell infiltration

Immune cell infiltration levels in normal human joints, osteoarthritic joints, and RA joints were calculated using the ssGSEA algorithm (20) [R software GSVA package (21)] based on the expression values in the mRNA dataset GSE55235. Differential analyses of the level of immune cell infiltration in RA and osteoarthritis were performed by the limma package. LogFC coefficients for the level of immune cell infiltration in RA relative to osteoarthritis were ranked from lowest to highest (selected to adjust for *p* values less than 0.05) (Supplementary Table 1).

### 2.4 Selection and enrichment analysis of DEGs related to the autophagy phenotype

The DEGs between the control and osteoarthritis groups and the control and RA groups in the mRNA dataset GSE55235 were determined to be associated with the autophagy phenotype using GeneCards (22).^3^ Intersecting genes were identified. Subsequently, an enrichment analysis was performed as described above (clusterProfiler package GO-KEGG analysis). Autophagy phenotype-related genes were extracted as follows: first, autophagy was searched on the GeneCards website, the results were downloaded, and the screening threshold was set. Genes with a relevance score higher than 30 were selected as autophagy phenotype-related genes.

### 2.5 Unsupervised consensus clustering of the level of immune cell infiltration in osteoarthritis and RA

For unsupervised consensus clustering, the expression values in the mRNA datasets GSE55235 and GSE153015 were downloaded and filtered for osteoarthritis and RA, respectively. Unsupervised consensus clustering is a commonly used method for resampling the original data that cluster a subset of the sampled data in multiple iterations to assess the probability that the samples belong to the same class (23).

We batch-corrected the osteoarthritis and RA datasets using the limma package to correct the data and PCA plots to describe the data before and after correction. The corrected data were subjected to unsupervised consensus clustering according to different groupings of osteoarthritis and RA. Suitable k values were selected according to the inflection point map and the data were divided into k clusters. The clustering screening results were displayed in the consensus matrix heat map. Finally, using the pheatmap package, the immune infiltration levels of immune cells in osteoarthritis and RA were heat-mapped separately.

### 2.6 Feature selection of RA and validation of the machine learning diagnosis model

To enhance the performance of the diagnostic model, combat-corrected GSE55235 and GSE153015 datasets were used for feature selection. Support vector machine-recursive feature elimination (SVM-RFE) (24) and LASSO logistic regression (LR) (25) were applied to screen candidate features. SVM-RFE is a machine learning method based on support vector machines that recursively removes features to identify the most informative variables, while LASSO LR selects variables by determining the *λ* value that minimizes the classification error. Both methods were used in combination, and the intersection of the features identified by the two approaches was taken as the final set of characteristic genes.

The LASSO LR model was implemented using the glmnet package (26), with 10-fold cross-validation to determine the optimal λ based on the minimum deviance criterion. The SVM-RFE model was implemented using the e1071 package, employing a linear kernel, 5-fold cross-validation, and a step size of 1 to recursively remove features while minimizing classification error. Model performance was evaluated using average accuracy and error rate.

The top 50 features identified by SVM-RFE were used to construct an additional SVM model, and the error rate and accuracy of this model were assessed. The feature genes identified by LASSO and SVM-RFE were then compared, and their intersection was selected for downstream validation. These final feature genes were further validated using ROC analysis in the original datasets (GSE55235 and GSE153015), and their expression differences across different immune subtype groupings were examined.

All analyses were performed in R 4.0.3 with a fixed random seed of 123 to ensure reproducibility.

### 2.7 PPI network construction

To further study the different biological mechanisms of RA compared with osteoarthritis, we selected RA as the disease group to compare the significant DEGs associated with osteoarthritis with the control group and predicted the PPI network^4^ of these genes. A comprehensive score of PPI ≥ 0.4 was considered to indicate reliable linkage for the construction of the PPI network. Then, Cytoscape software (27) (version 3.8.2) was adopted for visualizing the regulatory relationship between genes. Meanwhile, the topological properties of the network were calculated for analysis.

### 2.8 Prediction of differentially expressed miRNA target genes and transcription factors regulated by characteristic genes

Human miRNA–target gene interaction data were downloaded from miRDB (28) (version 5.0)^5^ and miRTarBase (29)^6^ databases. The miRDB data represented miRNA–refseq ID interaction pairs. The refseq IDs were converted into gene symbols with the hg38 annotation file, and the target gene sets with important miRNA characteristics were screened out. The miRNA–target gene interaction pairs from the two databases were intersected with the significant GSE72564 miRNA DEGs. There were 21 intersecting miRNAs among the interaction pairs derived from the miRDB database and 5 in the miRTarBase data. These 5 miRNAs were present in the results from both databases. The miRNA–target gene interaction pairs from the two databases were analyzed after combat with the two mRNA datasets in the previous article. The obtained sets of significant DEGs were subsequently intersected, and a total of four intersecting mRNAs (target genes) were found. The two results were combined to map the miRNA–mRNA (target–gene) network.

The Enrichr online tool (30)^7^ based on the TRANSFAC and JASPAR PWMs databases (31) was used to screen and analyze transcription factors that might have regulatory effects on the identified genes.

### 2.9 Immune cell correlation analysis and drug sensitivity analysis of characteristic genes

The association between the expression values of the characteristic variables and the level of immune cells (*R* > 0.3/*R* < −0.3 and *p* < 0.05) was analyzed in RA and osteoarthritis, respectively, by selecting the intersecting characteristic variables identified by LASSO and SVM-REF methods. The relationship was plotted in Sankey diagrams.

A drug sensitivity analysis was conducted using the CellMiner database (32) cell line IC50 data. Multiple FDA-approved, clinically tested drug molecules were screened for their therapeutic potential in relation to the screened genes. The correlations between gene expression levels and the IC50 of significantly relevant compounds were plotted (*R* > 0.3, *p* < 0.05).

### 2.10 Cell culture and treatment

Human fibroblast-like synoviocytes derived from rheumatoid arthritis patients (HFLS-RA; Catalog No. HTX1835) were purchased from Otwo Biotech (Shenzhen), China. The cells were shipped in a T25 flask containing approximately 2 × 10^6^ viable cells and cultured in fibroblast complete growth medium as recommended by the manufacturer. Cells were maintained at 37 °C in a humidified incubator with 5% CO_2_. The culture medium was refreshed every 2–3 days, and cells between passages 3 and 6 were used for all experiments to ensure phenotypic stability. During the logarithmic growth phase, HFLS-RA cells were detached using 0.25% trypsin–EDTA, counted, and evenly seeded into 6-well plates. Upon reaching approximately 70% confluence, cells were stimulated with 5 ng/mL recombinant human TNF-α (Servicebio, China) to establish an *in vitro* rheumatoid arthritis model. Cells were harvested at designated time points: 0 h, 6 h, 12 h, and 24 h post-treatment for downstream analyses.

### 2.11 Enzyme-linked immunosorbent assay

For the ELISA analysis, the supernatants were utilized and processed using commercially available kits for IL-1β, IL-6, and IL-8 (Servicebio, China), following the manufacturer’s instructions.

### 2.12 RT-qPCR

Total RNA was extracted from the clinical samples and cells using the TRIzol reagent (Servicebio, China). Subsequently, cDNA was synthesized using the PrimeScript RT Reagent Kit (Thermo Fisher Scientific, USA), and qPCR experiments were carried out using the SYBR Premix ExTaq kit (Roche, Switzerland). GAPDH was selected as the internal reference gene, and the 2^−ΔΔCt^ method was employed to normalize the gene expression levels. The primer sequences used in this study are listed in Table 1.

**Table 1:** The primer sequence used in this study.

Gene	Sequence (5′–3′)
GAPDH	F:GGAAGCTTGTCATCAATGGAAATC
	R:TGATGACCCTTTTGGCTCCC
LAP3	F:ACAGGTGCCATGGATGTAGC
	R:CTGTTTCAATGCTGGCCTCG
MAGED1	F:TGGAGATGAGGCTGTGTCTG
	R:ACTGGCTGTACCACCAGGAC
EPYC	F:GCCTCAACTTCGAGAGCTTGT
	R:GGCTCGTAGATTTTCTGGGAGT

### 2.13 Statistical analyses

The statistical analyses of the present study were based on R software (version 3.6.3) and GraphPad Prism 9 (version 9.5.1). *p* values were calculated using BH correction for microarray data in the analysis of variance and clusterProfiler package calculation function and pathway enrichment analysis. The gene expression levels detected via ELISA, RT-qPCR was compared with one-way ANOVA. Otherwise, the Mann–Whitney U test was used. A two-sided *p* < 0.05 indicated significance (* = <0.05, ** = <0.01, *** = <0.001, **** = <0.0001).

## 3 Results

### 3.1 Data preprocessing and screening of significant expression variables

RA microarray expression data and platform files were downloaded via the R GEOquery package (Table 2), and all microarray data were preprocessed for limma difference analysis after standardization and batch-effect correction.

**Table 2:** Information on microarray datasets obtained from Gene Expression Omnibus.

GEO dataset	Platform	Organism	Number of samples
GSE55235	GPL96 Affymetrix Human Genome U133A Array	*Homo sapiens*	Control = healthy joint (10); case 01 = osteoarthritic joint (10); case 02 = RA joint (10)
GSE153015	GPL570 Affymetrix Human Genome U133 Plus 2.0 Array	*Homo sapiens*	Control = osteoarthritic joint (4); case 02 = RA joint (4)
GSE72564	GPL20870 Qiagen Human miRNome miScript miRNA PCR arrays	*Homo sapiens*	Control = osteoarthritic joint (4); case 02 = RA joint (4)

RA, rheumatoid arthritis.

**Figure 1:**
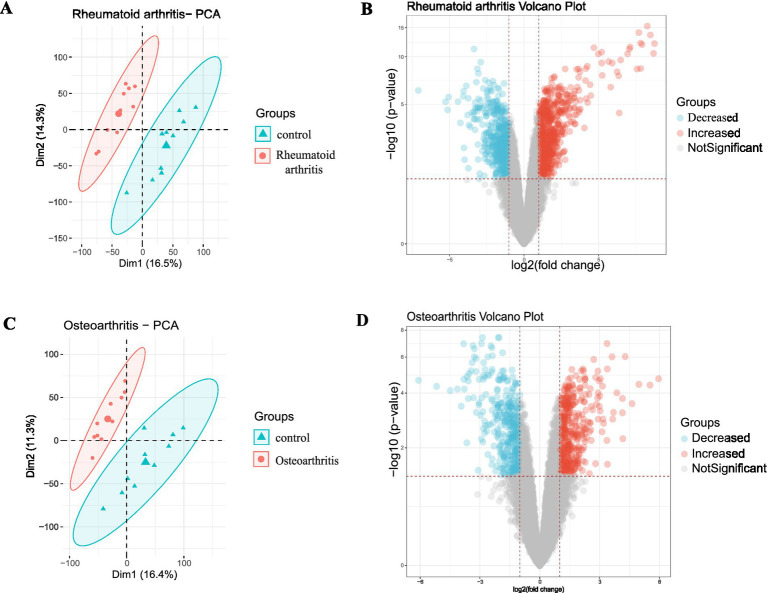
Batch effects and difference analysis of the osteoarthritis and RA groups. **(A)** Batch effect results of the RA group (untreated). **(B)** Volcano plot representing the differentially expressed mRNAs in the RA group; red denotes upregulation and blue stands for downregulation. **(C)** Batch effect results of the secondary osteoarthritis group (untreated). **(D)** Volcano plot showing the differentially expressed mRNAs in the osteoarthritis group; red stands for upregulation, and blue represents downregulation. RA: Rheumatoid arthritis.

The mRNA data were obtained from the GSE55235 dataset, and differences between the control and the osteoarthritis group and the control and the RA group were analyzed. The batch effects in the osteoarthritis and RA groups were identified by PCA (Figures 1A,C), and a volcano plot was used to visualize the DEGs (Figures 1B,D).

In the osteoarthritis group, 773 differentially expressed mRNA were obtained, 405 significantly upregulated and 368 significantly downregulated. In the RA group, 1,201 differentially expressed mRNA were obtained, 675 significantly upregulated and 526 significantly downregulated.

**Figure 2:**
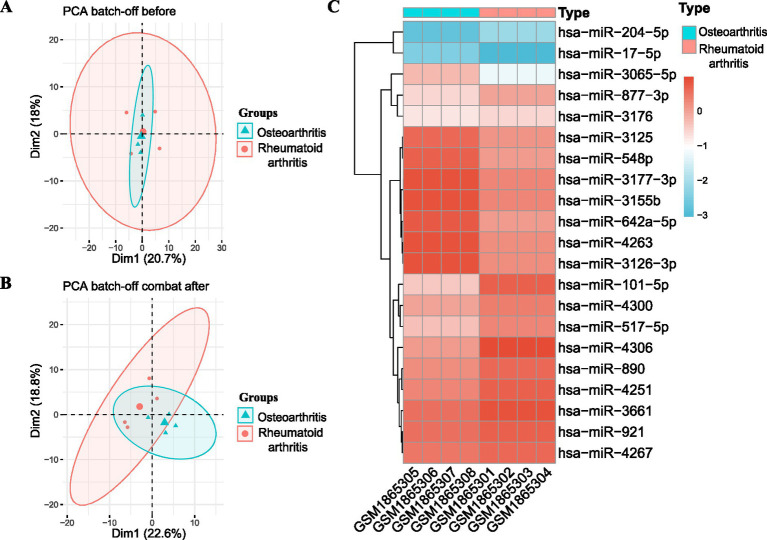
Batch-effect correction and difference analysis of miRNAs in the osteoarthritis and RA groups. **(A)** miRNA results before batch-effect correction in the osteoarthritis and RA groups. **(B)** miRNA results after batch-effect correction in the osteoarthritis and RA groups. **(C)** miRNA heatmap of the RA and osteoarthritis group comparison showing differentially expressed miRNAs in the osteoarthritis group; red suggests high expression levels, and blue stands for low expression levels. RA: Rheumatoid arthritis.

The miRNA dataset was derived from the GSE72564 dataset. The PCA images before and after batching are shown (Figures 2A,B). The heatmap shows differentially expressed miRNAs (Figure 2C). Finally, 21 differentially expressed miRNAs were obtained, 12 significantly upregulated and 9 significantly downregulated.

### 3.2 Function and pathway enrichment analysis

**Figure 3:**
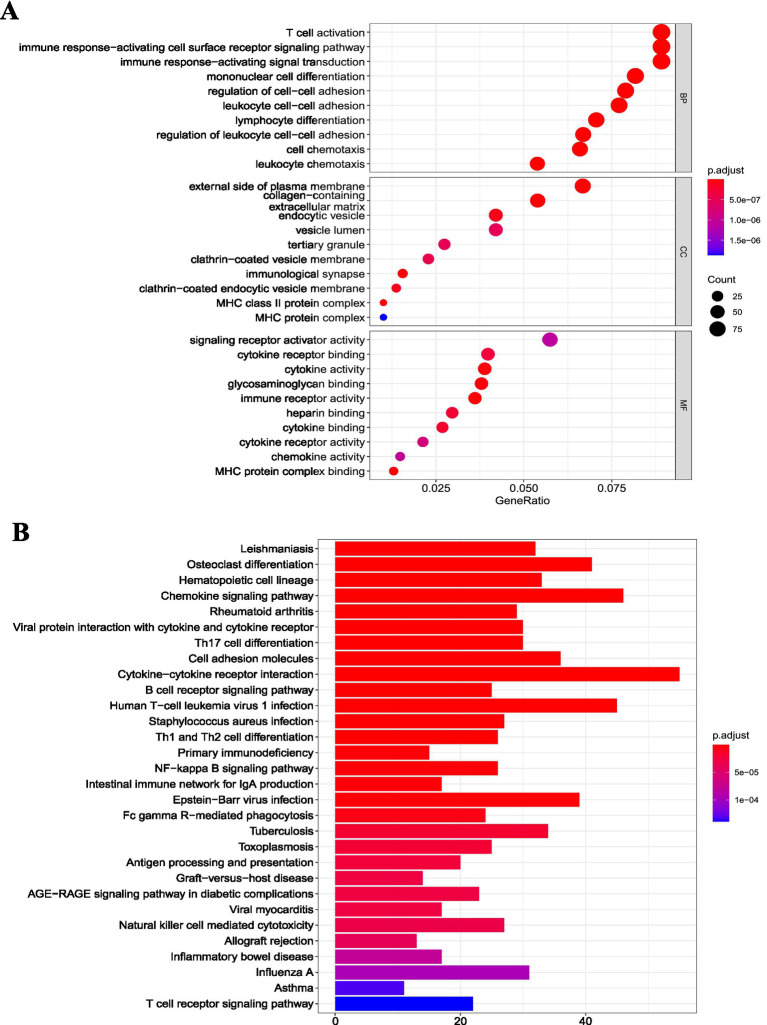
Functional pathway enrichment analysis of differentially expressed mRNAs in the rheumatoid arthritis group. **(A)** GO functional enrichment analysis of differentially expressed mRNAs in the rheumatoid arthritis group; **(B)** KEGG pathway enrichment analysis of differentially expressed mRNAs in the rheumatoid arthritis group. GO: Gene Ontology; KEGG: Kyoto Encyclopedia of Genes and Genomes; RA: rheumatoid arthritis.

**Figure 4:**
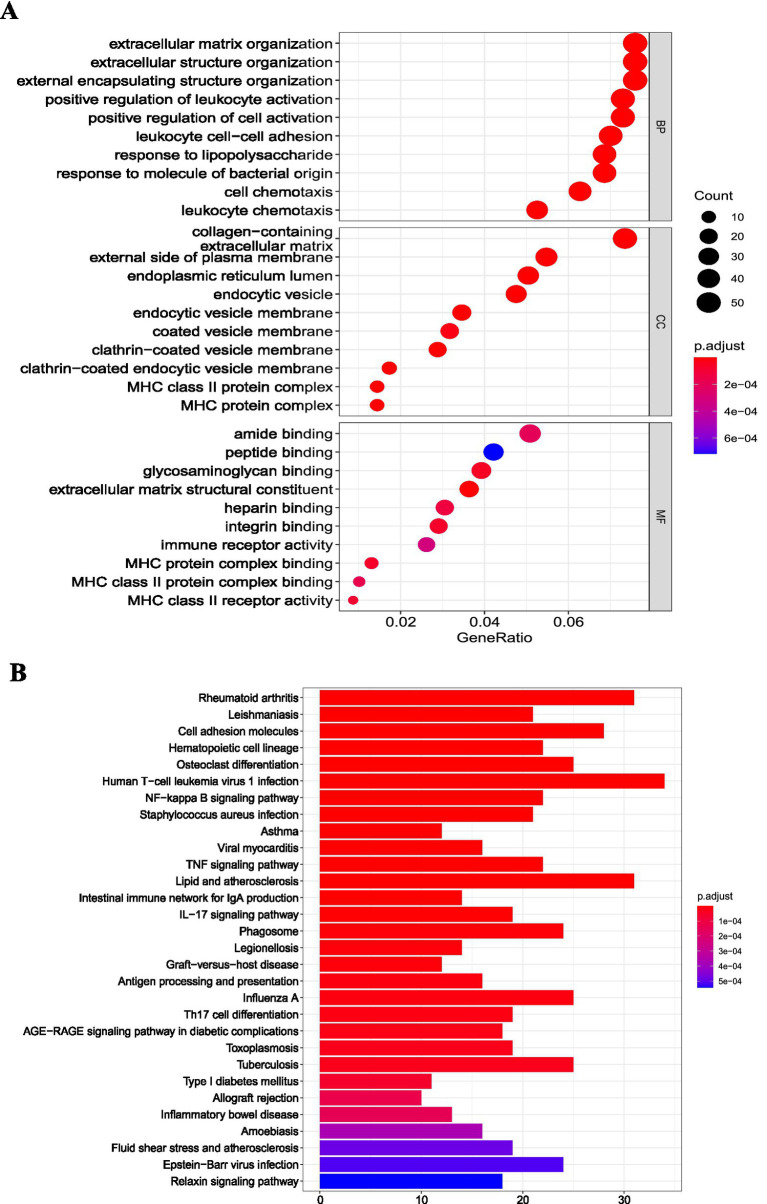
Functional pathway enrichment analysis of differentially expressed mRNAs in the osteoarthritis group. **(A)** GO functional enrichment analysis of differentially expressed mRNAs in the osteoarthritis group; **(B)** KEGG pathway enrichment analysis of differentially expressed mRNAs in the osteoarthritis group. GO: Gene Ontology; KEGG: Kyoto Encyclopedia of Genes and Genomes; RA: rheumatoid arthritis.

**Figure 5:**
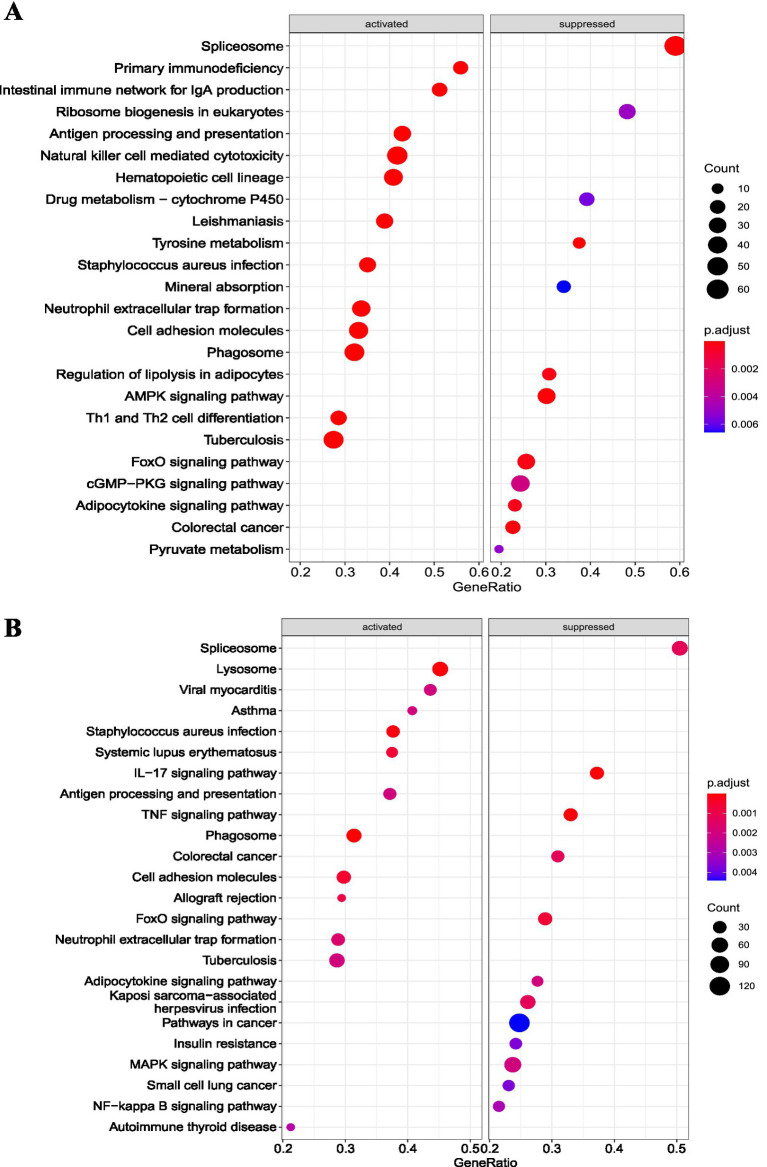
GSEA pathway enrichment analysis of differentially expressed mRNAs in the rheumatoid arthritis group and the osteoarthritis group. **(A)** GSEA pathway enrichment results of the DEGs in the rheumatoid arthritis group; **(B)** GSEA pathway enrichment results of the DEGs in the osteoarthritis group. GSEA: Gene Set Enrichment Analysis.

We employed the clusterProfiler package to enrich the DEGs of the osteoarthritis and RA groups in the GSE55235 dataset. In the RA group, GO (Figure 3A) and KEGG enrichment pathway (Figure 3B) analyses showed that the DEGs were mainly associated with immune-related pathways. In the osteoarthritis group, GO (Figure 4A) and KEGG enrichment pathway (Figure 4B) analyses showed that the DEGs were mainly associated with extracellular tissue-related pathways. GSEA results further supported these findings in the RA group (Figure 5A) and the osteoarthritis group (Figure 5B). GSEA used “c2.cp.v7.4.symbols.gmt” as the reference gene set. In addition, false discovery rates (FDR) < 0.25 and *p* values <0.05 indicated significant enrichment.

### 3.3 Calculation and cluster analysis of immune cell infiltration level

**Figure 6:**
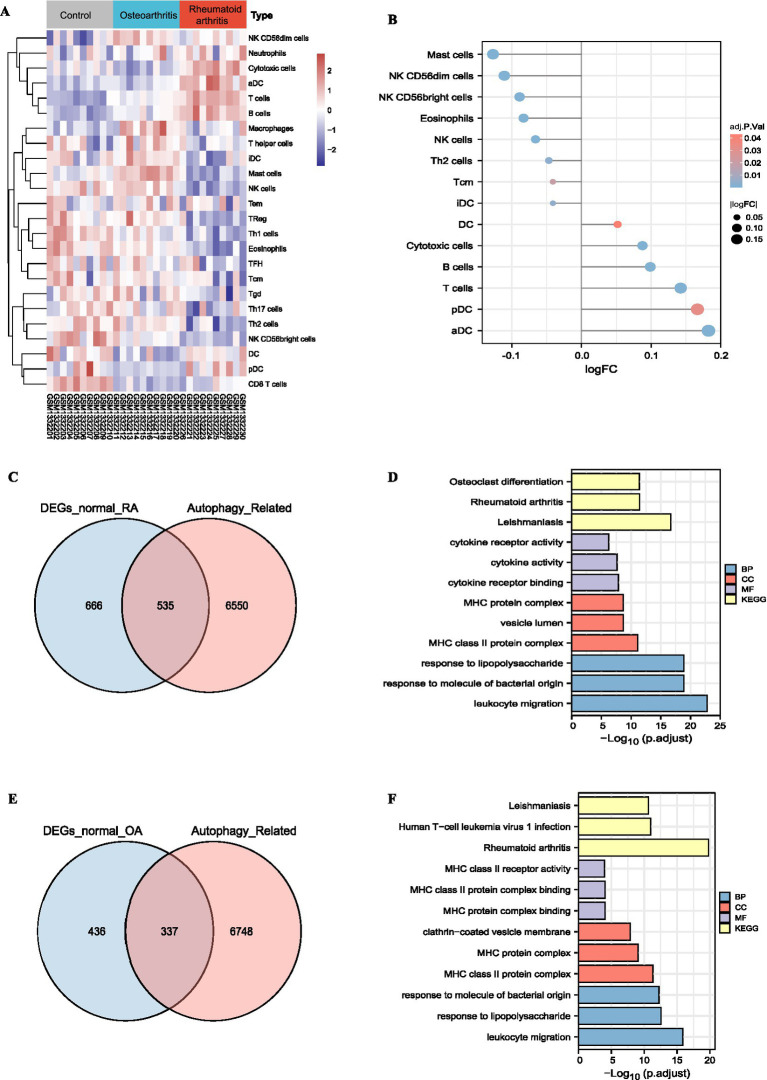
The correlation of osteoarthritis and RA with the level of immune cell infiltration and autophagy: **(A)** Immune cell infiltration levels in normal, osteoarthritis, and RA joints. **(B)** The intersection of significant DEGs in osteoarthritis and autophagy-related genes. **(C)** Enrichment analysis of significant DEGs in osteoarthritis and autophagy-related genes. **(D)** The intersection of significant DEGs in RA and autophagy-related genes; **(E)** Enrichment analysis of significant DEGs in RA and autophagy-related genes. **(F)** Functional enrichment analysis of the intersecting genes between significant DEGs in osteoarthritis and autophagy-related genes. RA: rheumatoid arthritis.

The immune infiltration levels of immune cells in normal human joints, osteoarthritis joints, and RA joints were calculated by ssGSEA algorithm 12 (R software GSVA package) according to the expression values of mRNA in the GSE55235 dataset (Figure 6A). Then, the level of immune cell infiltration between RA and osteoarthritis was compared. The analysis method used the limma package. The level of immune cell infiltration in RA was lower in relative to the logFC coefficient of osteoarthritis. Arrange to high (choose to adjust *p*-value less than 0.05) (Figure 6B). Next, we further analyzed the overlap between differentially expressed genes (DEGs) and autophagy-related genes in rheumatoid arthritis (RA) compared with normal controls. A total of 666 overlapping genes were identified between the 1,201 DEGs and 535 autophagy-related genes (Figure 6C). Functional enrichment analysis of these intersecting genes revealed that they were mainly involved in cytokine activity, leukocyte migration, and other inflammation-related biological processes (Figure 6D). Similarly, overlap analysis between the DEGs in osteoarthritis (OA) and autophagy-related genes identified 436 intersecting genes between the 773 DEGs and 337 autophagy-related genes (Figure 6E). Functional enrichment analysis further highlighted the significance of MHC-related functions and their important roles in immune responses in OA (Figure 6F).

**Figure 7:**
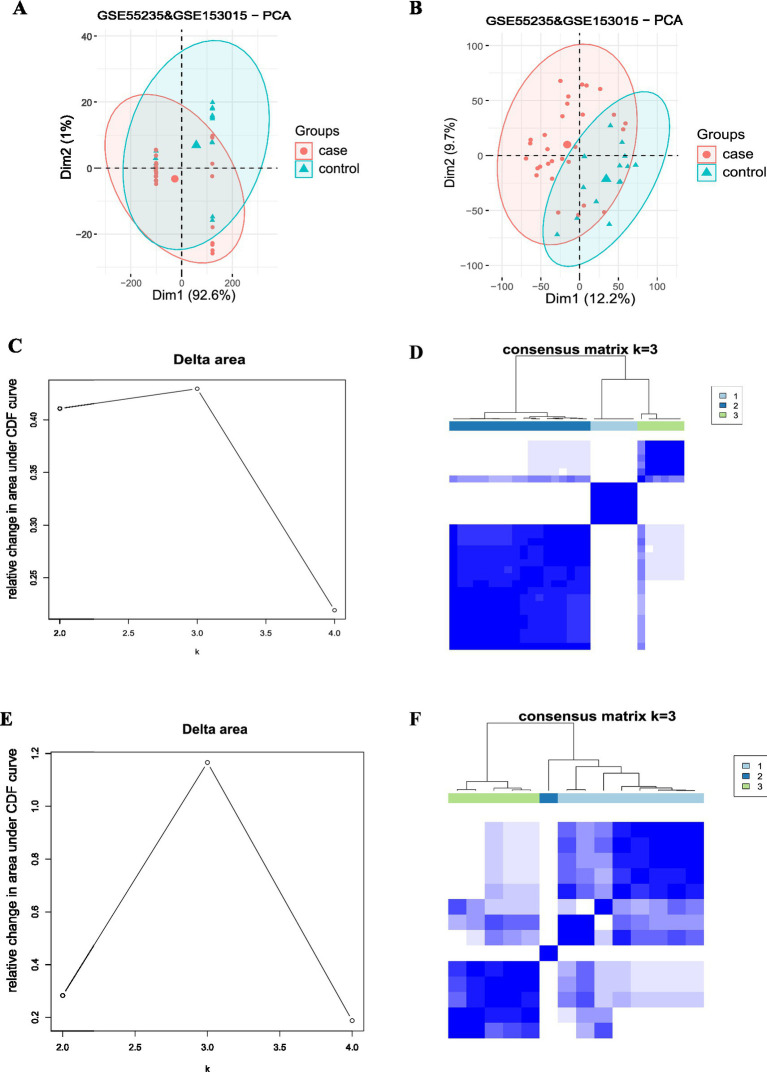
Unsupervised consensus clustering of osteoarthritis and RA: **(A)** GSE55235 and GSE153015 osteoarthritis and RA datasets before batch-effect correction. **(B)** GSE55235 and GSE153015 osteoarthritis and RA datasets after batch-effect correction. **(C)** The RA dataset was selected according to the inflection point plot (maximum at *k* = 3). **(D)** The RA dataset was divided into three clusters, and the clustering results were obtained by consensus matrix. The osteoarthritis dataset was selected using the inflection point graph with a suitable *k* value (maximum at *k* = 3). **(E)** The RA data were classified into 3 clusters, and the clustering results were obtained by consensus matrix. **(F)** The osteoarthritis data were categorized into 3 clusters, with the clustering results being denoted in the consensus matrix heatmap. RA: Rheumatoid arthritis.

**Figure 8:**
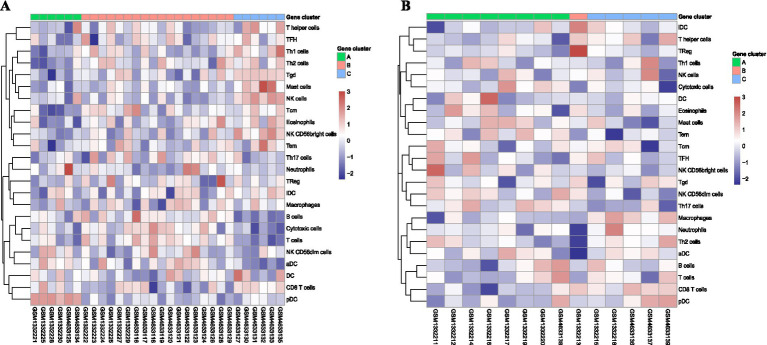
Heatmap of the immune cell infiltration level in osteoarthritis and RA: **(A)** Heatmap of the level of immune cell infiltration in RA; **(B)** Heatmap of the immune cell infiltration level in osteoarthritis. RA: rheumatoid arthritis.

The mRNA expression values were downloaded for datasets GSE55235 and GSE153015, and the osteoarthritis and RA data from the two datasets were batch-effect corrected based on the combat method in the limma package. The data before and after correction were depicted using PCA (before correction: Figure 7A; after correction: Figure 7B). The corrected data were grouped by osteoarthritis and RA, and unsupervised consensus clustering was performed for each group. Suitable k values were selected using an inflection point diagram. Both inflection point maps had the largest inflection point when *k* = 3; thus, *k* = 3 was selected for clustering (RA: Figure 7C; osteoarthritis: Figure 7E). Then, the data were classified into 3 clusters, with clustering results being displayed in a consensus matrix heatmap (RA: Figure 7D; osteoarthritis: Figure 7F). Finally, the immune cell infiltration levels in osteoarthritis and RA were visualized separately using the pheatmap package (RA: Figure 8A; osteoarthritis: Figure 8B).

### 3.4 Feature selection of RA and validation of the machine learning diagnosis model

**Figure 9:**
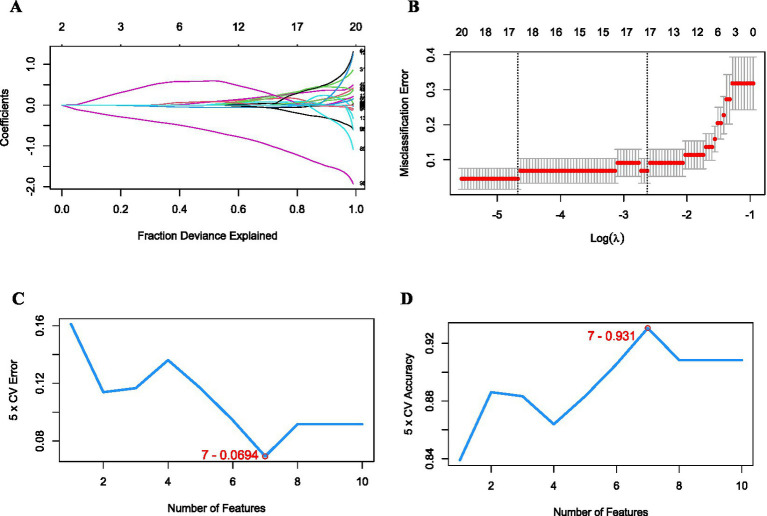
Feature selection of osteoarthritis and RA classification and machine learning diagnosis model construction. **(A)** The LASSO LR feature selection method screens the characteristic mRNAs most relevant to the classification of osteoarthritis and RA among the DEGs. **(B)** The LASSO LR feature selection method screening process to determine the best lambda. **(C)** SVM model error rate (lowest point of error rate: 0.0694). **(D)** SVM model accuracy rate (highest point of accuracy: 0.931). RA: rheumatoid arthritis; LASSO LR: LASSO logistic regression; SVM: support vector machine.

The diagnostic model was constructed using combat-corrected GSE55235 and GSE153015 data. SVM-RFE and LASSO logistic regression were adopted for screening the characteristic variables. We employed LASSO LR (Figures 9A,B) and SVM, two machine learning methods, to screen mRNA feature variables and selected feature variables identified by both methods as important mRNA feature variables. The first 50 variables in SVM were selected for a second SVM model construction, and the error (Figure 9C) and accuracy (the highest point 0.931) rates (Figure 9D) were determined. The lowest point of error rate, 0.0694, was selected as the basis for variable screening to confirm that SVM reliably screened the 7 characteristic variables.

**Figure 10:**
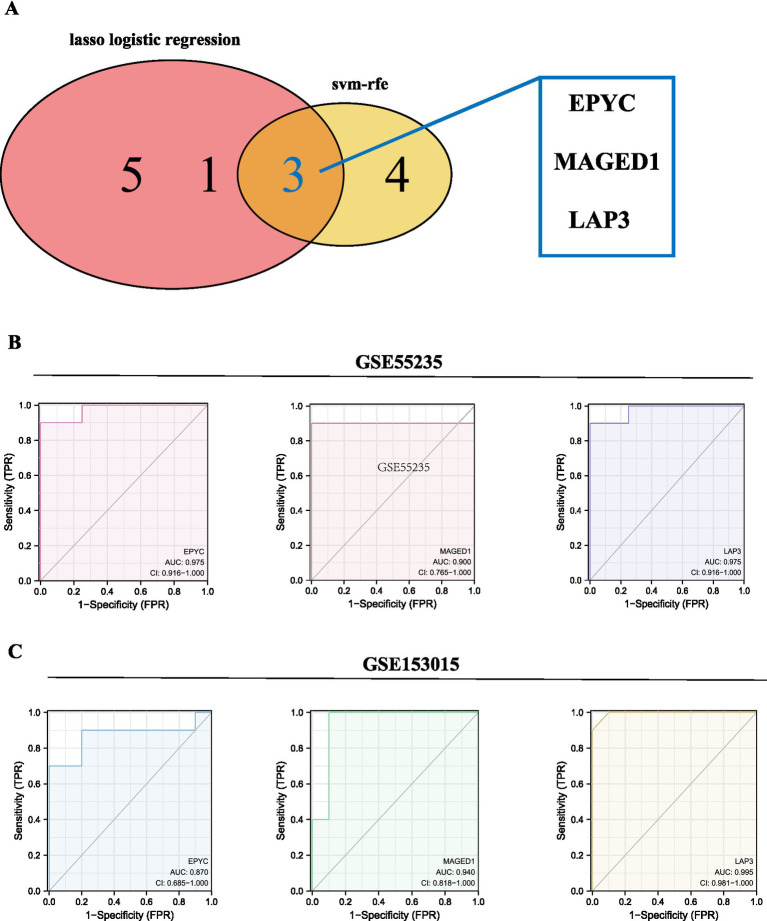
Feature selection intersection and ROC verification of osteoarthritis and RA classification: **(A)** LASSO LR and SVM feature selection revealed three feature genes, EPYC, MAGED1, and LAP3. **(B)** ROC verification using the GSE55235 original dataset; the AUC values of the three feature genes are all above 0.9 in the two datasets. **(C)** ROC verification using the GSE153015 original dataset; the AUC values of the three feature genes are all above 0.85 in the two datasets. RA: rheumatoid arthritis; LASSO LR: LASSO logistic regression; SVM: support vector machine.

**Figure 11:**
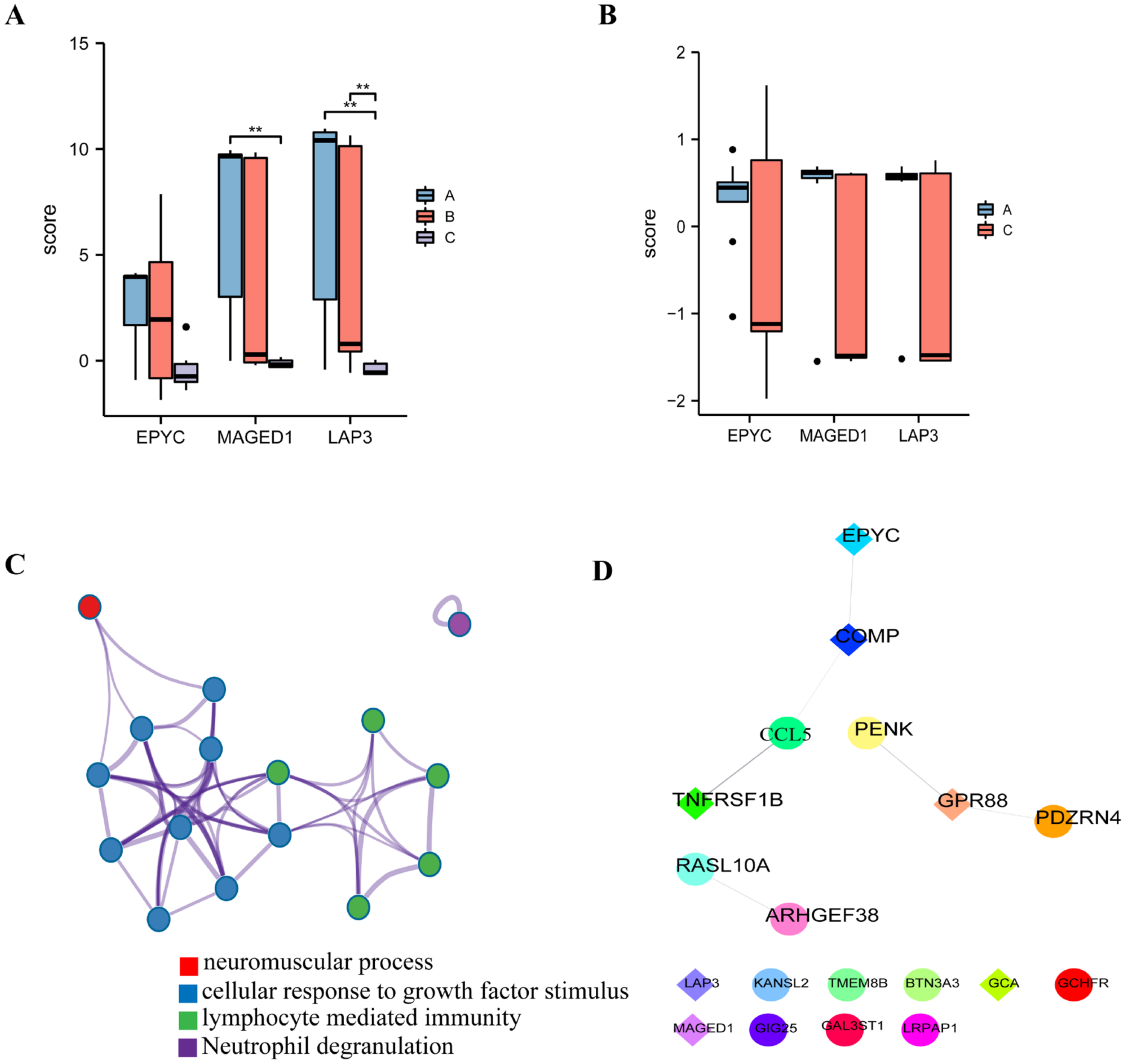
Differences in the expression levels of the characteristic genes in different immune subtype groups: **(A)** Differences in the expression levels of characteristic genes in different immune subtypes of RA; **(B)** Differences in the expression levels of characteristic genes in different immune subtypes of osteoarthritis. **(C)** RA in relative to the control group; the significant DEGs of inflammatory disease were analyzed by Metascape. **(D)** Comparison of the significant DEGs in the RA and osteoarthritis groups; a PPI network analysis was performed. RA: rheumatoid arthritis.

LASSO LR screened out 18 mRNA characteristic variables, and SVM screened 7. The intersection set was constructed with 3 characteristic variable mRNAs (Figure 10A). The characteristic variables found by LASSO and SVM-REF were compared to identify intersecting mRNAs. Three feature genes (EPYC, MAGED1, and LAP3) were selected for ROC verification using the original datasets, GSE55235 and GSE153015. The AUC values of the three feature genes in the two datasets exceeded 0.85 (Figures 10B,C). In addition, differences in the expression levels of characteristic genes in different immune subtype groups were evaluated and differed significantly across immune subtypes. Due to the sample size, only MAGED1 had *p* values below 0.05 in the RA immune subtype group. The immune subtype A of MAGED1 was significantly different from the immune subtype C (*p* < 0.05) (Figure 11A), while LAP3 was in the third. There existed significant differences among all immune subtypes (*p* < 0.05) (Figure 11A). By contrast, although an obvious trend was present in the osteoarthritis group, the three core genes did not demonstrate statistically significant differences (Figure 11B).

### 3.5 PPI network construction and prediction of miRNA target genes and transcription factors

To further investigate the different biological mechanisms of RA compared with osteoarthritis, we selected RA as the disease group for comparison with the significant DEGs in the osteoarthritis group. We then predicted the functional enrichment of the DEGs using the Metascape website. As shown in the figure, the significant DEGs were mostly enriched in pathways such as cellular response to growth factor stimulus and lymphocyte-mediated immunity (Figure 11C). We predicted the PPI network that these genes might construct. The proteins corresponding to these significant DEGs did not demonstrate a clear network. The comprehensive score of the PPI network was <0.4, and the reliability of the connection relationship was poor (Figure 11D).

**Figure 12:**
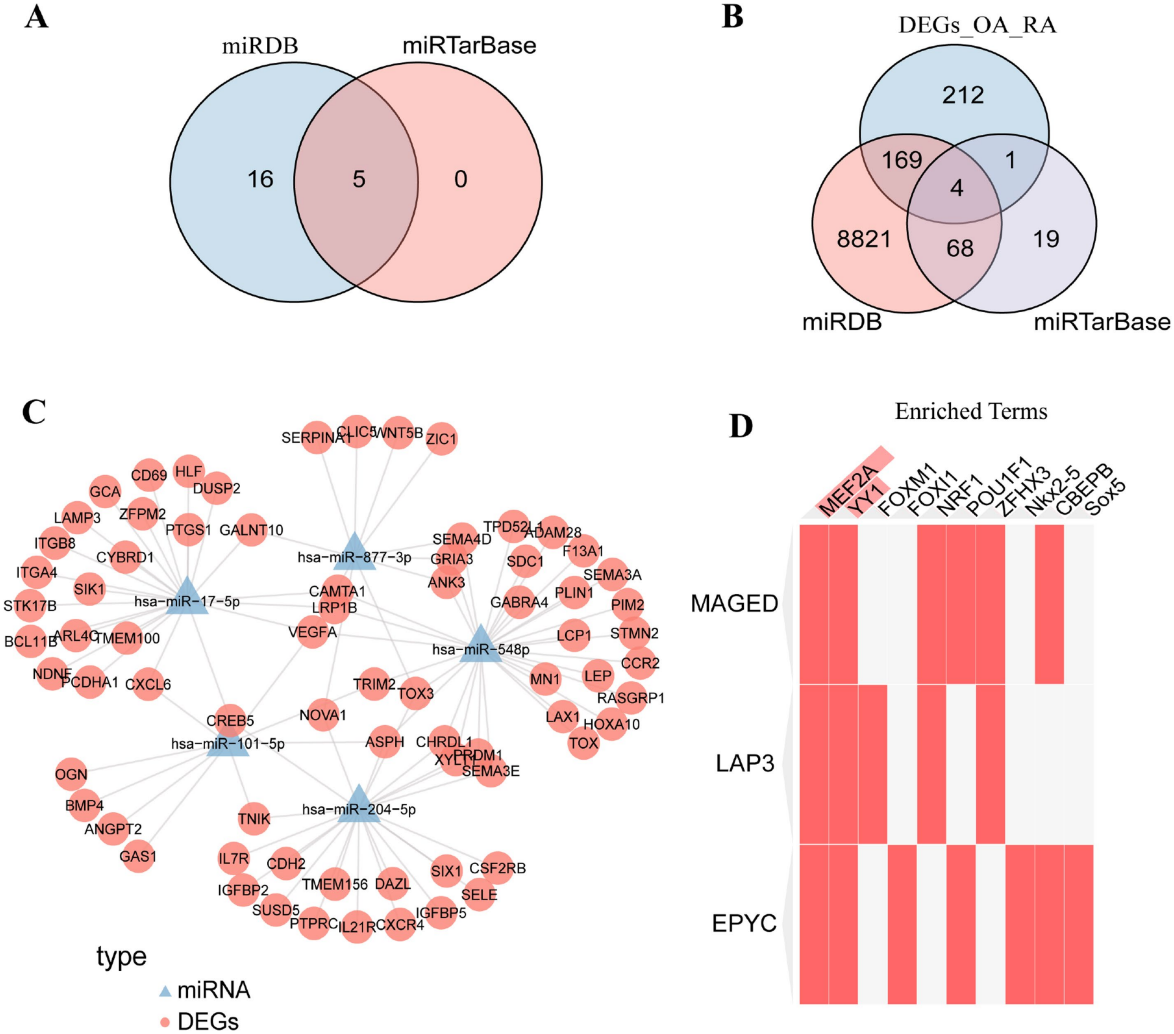
MiRNA–target–gene regulatory network and transcription factor prediction **(A)** miRNAs intersecting with the significant DEGs in the interaction pairs derived from miRDB and miRTarBase. **(B)** mRNA intersecting with the significant DEGs in the interaction pairs derived from miRDB and miRTarBase. **(C)** iRNA–target–gene regulation network. **(D)** Enrichr online tool (based on TRANSFAC and JASPAR PWMs database results) screened and analyzed transcription factors that might regulate characteristic genes.

Human miRNA–target gene interaction data were downloaded from the miRDB (version 5.0) (see text footnote 5) and miRTarBase (see text footnote 6) databases. The miRNA target gene interaction pairs from the two databases intersected with the significantly differentially expressed miRNAs in GSE72564. Twenty-one miRNAs were intersected in the miRDB database and 5 miRNAs in miRTarBase; these 5 were shared across the two databases (Figure 12A). Then, the miRNA target gene interaction pairs from the two databases were intersected with the significant DEGs obtained from the post-combat analysis of the two mRNA datasets in the previous experiments, and a total of four intersected mRNAs (target genes) were found (Figure 12B). The miRNA–mRNA (target gene) network was subsequently plotted (Figure 12C).

Enrichr (see text footnote 7) was used to screen and analyze the transcription factors that potentially regulate the signature genes based on TRANSFAC and JASPAR PWMs database results; MEF2A and YY1 were most likely to regulate the three signature genes (Figure 12D).

**Figure 13:**
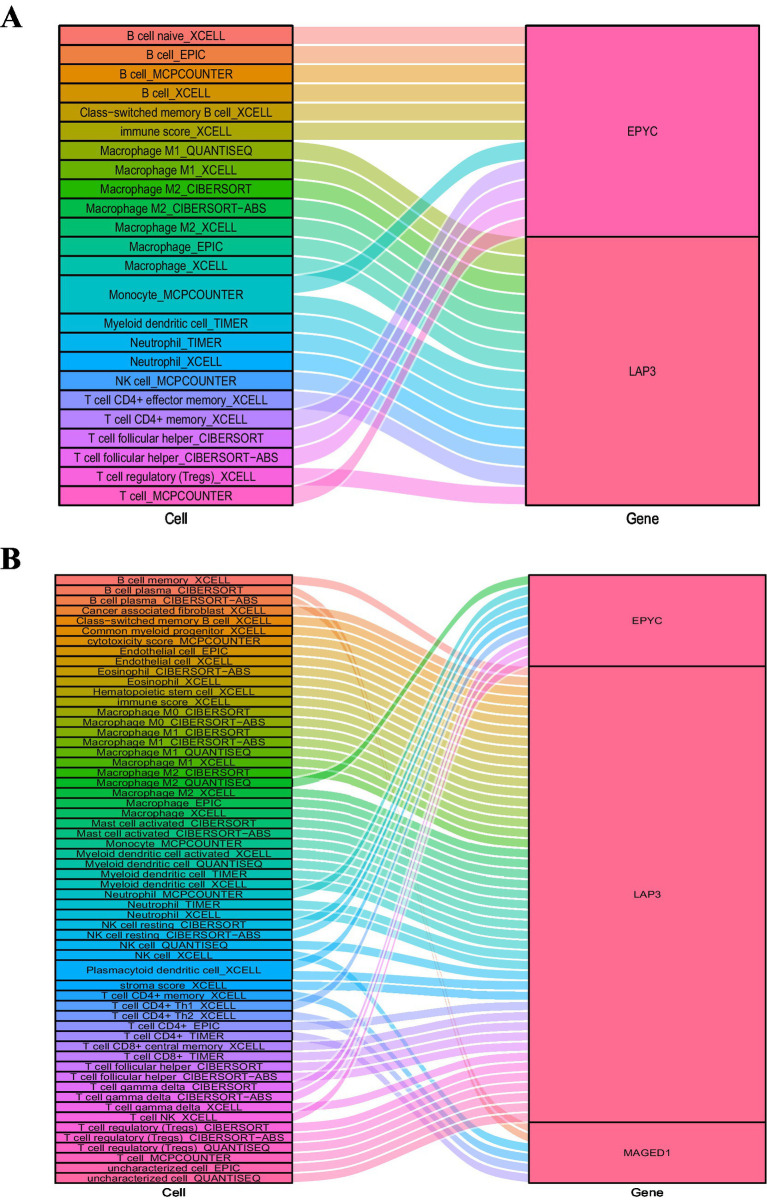
Relationship between characteristic genes and the level of immune cell infiltration. **(A)** The relationship between characteristic genes and the level of immune cell infiltration in RA. **(B)** The correlation between characteristic genes and the level of immune cell infiltration in osteoarthritis. RA: Rheumatoid arthritis.

The intersection characteristic variables identified by LASSO and an alpha of <0.05 on both sides were considered statistically significant. SVM-REF methods were selected to explore the correlation between the expression value of characteristic variables and the level of immune cells in RA and osteoarthritis (*R* > 0.3/*R* < −0.3 and *p* < 0.05). The relationship between the two was plotted by Sankey plots (Figures 13A,B). In RA, EPYC and LAP3 were related to most immune cells (Figure 13A), while in osteoarthritis, this was only true for LAP3 (Figure 13B).

Drug sensitivity analysis was based on CellMiner database cell line IC50 data. Multiple FDA-approved, clinically tested drug molecules were screened for their therapeutic potential in relation to the screened genes. The correlations between gene expression levels and the IC50 of significantly relevant compounds were plotted (*R* > 0.3, *p* < 0.05).

**Figure 14:**
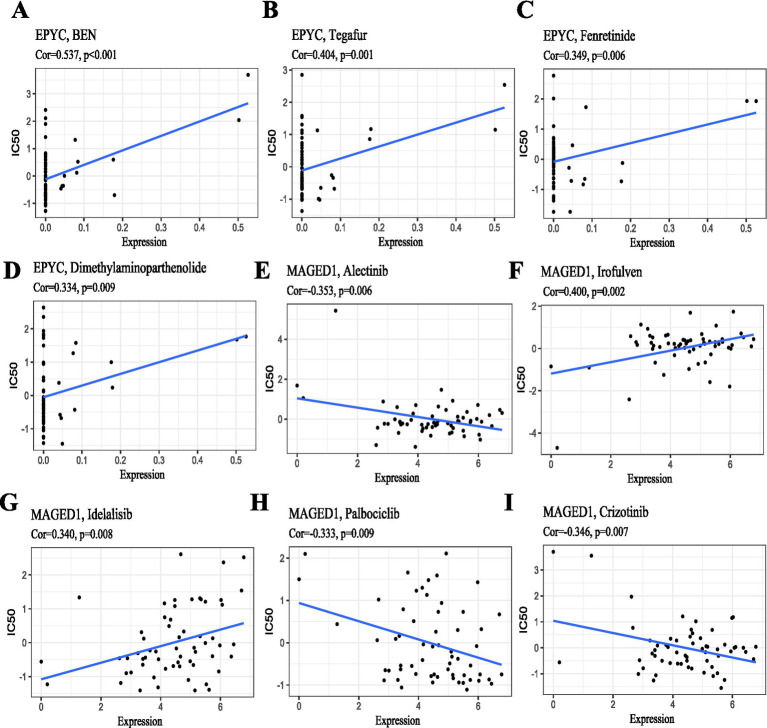
Drug sensitivity analysis of characteristic genes. Plotting the relationship between gene expression levels and the IC_50_ of compounds significantly associated with characteristic genes based on CellMiner database cell line IC_50_ data. **(A–D)** Correlation between EPYC expression and the IC_50_ of BEN, tegafur, fenretinide, and dimethylaminoparthenolide, respectively. **(E–I)** Correlation between MAGED1 expression and the IC_50_ of alectinib, irofulven, idelalisib, palbociclib, and crizotinib, respectively.

Our screening identified several compounds statistically associated with EPYC expression, including BEN, tegafur, fenretinide, and dimethylaminoparthenolide (Figures 14A–D); those with therapeutic potential against MAGED1 were alectinib, irofulven, idelalisib, Palbociclib, and crizotinib (Figures 14E–I).

### 3.6 Impact of TNF-α induction on the levels of IL-1β, IL-6, and IL-8 in HFLS-RA cells

**Figure 15:**
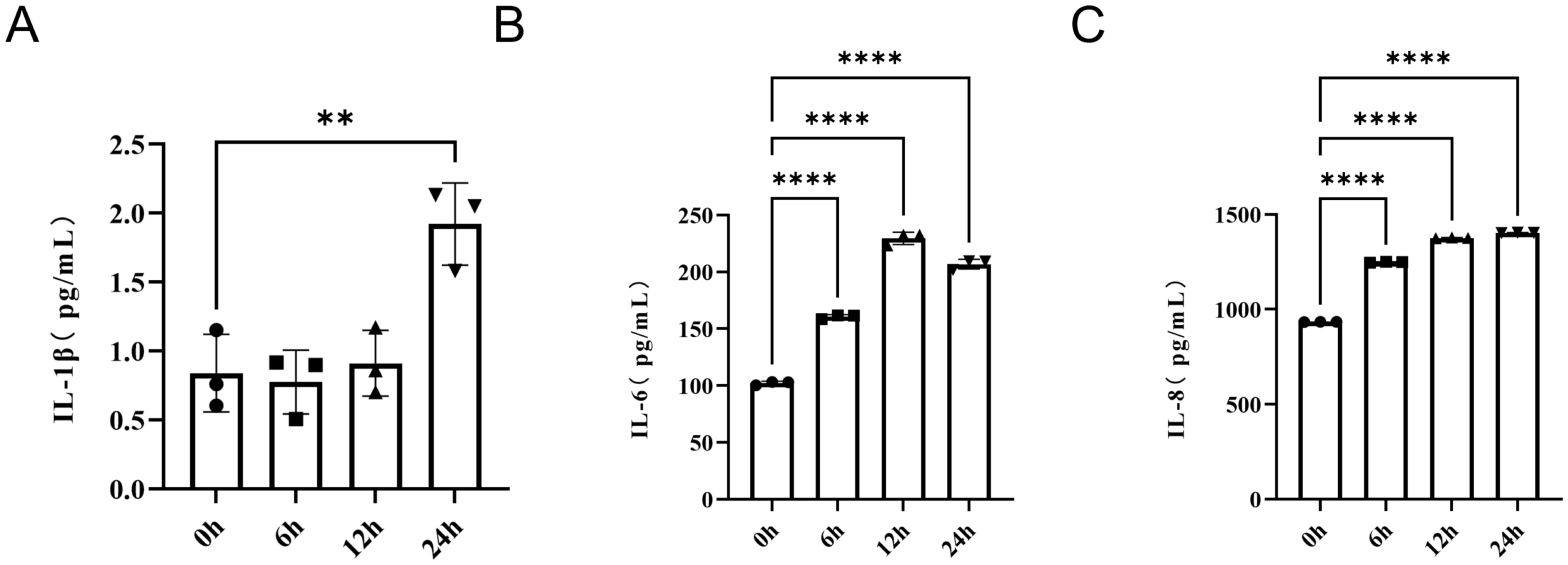
Effects on IL-1β **(A)**, IL-6 **(B)**, and IL-8 **(C)** levels in HFLS-RA cells induced by TNF-α (5 ng/mL). *n* = 3; vs. 0 h, ^**^p < 0.01; ^****^p < 0.0001.

In comparison to the 0-h group, IL-1β levels were significantly elevated at 24-h (*p* < 0.01) (Figure 15A). The levels of IL-6, when compared to the 0-h group, showed a significant increase in the 6-h, 12-h, and 24-h groups (*p* < 0.0001) (Figure 15B). Similarly, the levels of IL-8, in comparison to the 0-h group, significantly increased in the 6-h, 12-h, and 24-h groups (*p* < 0.0001) (Figure 15C).

### 3.7 qRT-PCR validation of hub genes

**Figure 16:**
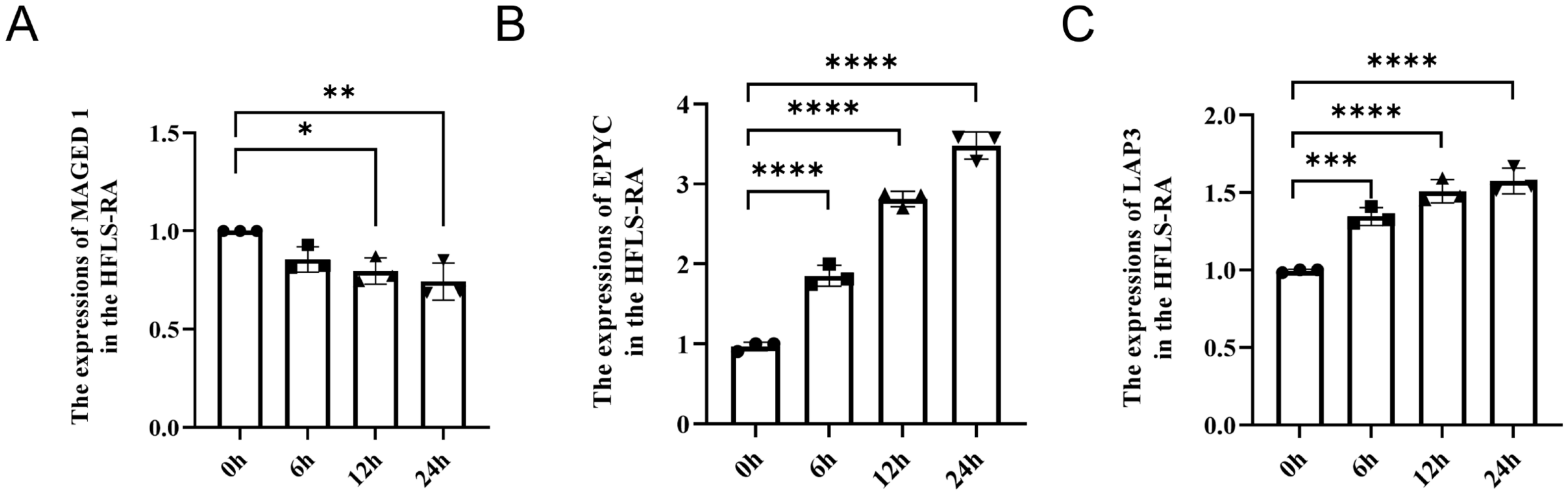
Quantitative analysis of MAGED1 **(A)**, EPYC **(B)**, and LAP3 **(C)** mRNA expression in HFLS-RA induced by TNF-α (5 ng/mL). *n* = 3; vs. 0 h, ^*^p < 0.05, ^**^p < 0.01, ***p < 0.001, ****p < 0.0001.

We assessed the relative expression of MAGED1, EPYC, and LAP3 in TNF-α (5 ng/mL)-induced HFLS-RA using qRT-PCR. When compared to the 0-h group, the mRNA expression of MAGED1 was significantly lower in the 12-h-induced group (*p* < 0.05) and significantly lower in the 24-h-induced group (*p* < 0.01) (Figure 16A). The mRNA expression of EPYC, when compared to the 0-h group, significantly increased in the 6-h, 12-h, and 24-h-induced groups (*p* < 0.0001) (Figure 16B). The mRNA expression of LAP3, when compared to the 0-h group, significantly increased in the 6-h-induced group (*p* < 0.001) and significantly increased in the 12-h and 24-h-induced groups (*p* < 0.0001) (Figure 16C).

## 4 Discussion

RA is characterized by chronic synovitis, which can lead to joint erosion and damage. Early detection and treatment of RA can prevent joint destruction and improve patient quality of life. Substantial advances have been made in RA treatment; however, the lack of early biomarkers often results in missed opportunities for timely intervention. Therefore, identifying effective biomarkers for RA remains of clear clinical importance.

In our study, we analyzed transcriptomic differences between the OA and RA groups. The OA group exhibited 773 differentially expressed mRNAs, including 405 upregulated and 368 downregulated, whereas the RA group had 1,201 differentially expressed mRNAs, with 675 upregulated and 526 downregulated. Comparing the two groups revealed 21 miRNAs, 12 upregulated and 9 downregulated. The grouping strategy adopted in this study was designed to enhance diagnostic specificity. While comparisons with healthy controls can identify disease-associated transcriptional changes, many alterations may reflect general inflammatory responses shared across joint disorders. Therefore, direct comparison between RA and OA allowed further refinement of candidate genes and identification of RA-associated signatures beyond common inflammatory mechanisms. Functional enrichment analysis showed that DEGs in the RA group were mainly associated with immune-related pathways, including T-cell activation, T-cell proliferation, MHC class II receptor activity, and Th1/Th2 cell differentiation (33). These pathways contribute to RA synovitis, which underlies arthritis and arthralgia.

Considering the functional enrichment results, we further evaluated immune cell infiltration using ssGSEA. In RA, NK CD56dim cells, B cells, T cells, activated dendritic cells (aDC), cytotoxic cells, and neutrophils were upregulated, whereas macrophages, iDCs, mast cells, NK cells, Tems, Tregs, Th1 cells, eosinophils, Tcms, Tgds, Th17 cells, Th2 cells, NK CD56bright cells, DCs, pDCs, and CD8+ T cells were downregulated. These discrepancies may reflect dataset heterogeneity and the limitations of computational deconvolution methods. T cells migrate into inflamed joints through rolling, arrest, spreading, crawling, and transmigration (34). Studies have shown that antibody Fc regions, type II collagen, cyclic citrullinated peptides appear in the serum of RA patients much earlier than the onset of disease, indicating impaired B cell tolerance (35–37). Macrophage and neutrophil infiltration occurs in the early stage of RA and might be related to bone and joint erosion (38). Th1, Th17, and Treg cells are present in the inflamed joint tissues of RA patients (39). Studies have shown that DCs cause arthritis by acting as antigen-presenting cells and promoting T-cell activation (40). It has been shown that NK cells could play a protective and pathogenic role in RA, but the data on the number of NK cells remain controversial in different studies (41–43). Therefore, these ssGSEA-based findings should be interpreted cautiously and considered exploratory.

Recent studies have further highlighted the heterogeneity of the RA synovial microenvironment. Single-cell and spatial analyses indicate that RA synovium consists of distinct cellular phenotypes with varying lymphoid, myeloid, and stromal activation states (44, 45). These differences are closely related to local immune activity, histologic features, and treatment response. In this context, the immune-related pathways enriched in our study align with current understanding that dysregulated immune cell interactions are central in RA synovitis. These findings also suggest that transcriptome-based estimates of immune infiltration reflect aggregate signals from heterogeneous tissues and should be interpreted with caution.

Recent work has emphasized the growing value of machine learning in RA biomarker discovery (46, 47). Given the clinical and biological heterogeneity of RA, conventional comparisons with healthy controls may detect many inflammation-related changes that are not specific to RA. In our study, direct comparison between RA and OA after the initial disease-versus-control analyses allowed refinement of candidate genes and focus on RA-specific molecular features. This design is clinically relevant, as the key challenge is distinguishing RA from other joint diseases rather than detecting general inflammation. Accordingly, EPYC, MAGED1, and LAP3 emerged as candidate markers to discriminate RA from OA, although their diagnostic utility requires validation in larger, independent cohorts.

The biological significance of the identified genes should be interpreted in the context of RA tissue heterogeneity (48). Stromal and immune cell states vary across disease stages and inflammatory subtypes. While EPYC, MAGED1, and LAP3 showed good discriminatory performance in our analyses and were supported by *in vitro* validation, their expression may still be influenced by tissue composition and inflammatory context. Future studies incorporating larger clinical cohorts with single-cell or spatial approaches will be important to clarify their roles in RA pathogenesis and diagnostic potential.

Using SVM-RFE and LASSO logistic regression, we identified three characteristic genes: LAP3, MAGED1, and EPYC. ROC analysis indicated that all three had good diagnostic performance. An in vitro RA model in HFLS-RA cells was established by TNF-α (5 ng/mL) stimulation, confirmed by IL-1β, IL-6, and IL-8 measurements. Following TNF-α induction, EPYC and LAP3 were upregulated, while MAGED1 was downregulated, consistent with bioinformatics predictions.

Some studies have reported a potential relationship between the three characteristic genes and the pathogenesis of RA. LAP3 presents significant upregulation in the disease due to hyperinflammation and can be regarded to be an underlying for anti-inflammatory drugs (49). Moreover, a recent study showed that plasma levels of LAP3 are obviously increased in the plasma of NAFLD patients (50). As of the vital M1 LAP members, LAP3 exerts multifunctional roles in tumor metastasis, for instance, stimulating cell proliferation and migration in glioma tumors (51, 52). LAP3 levels are increased significantly in patients with NAFLD patients compared with healthy controls (53). In addition, the mRNA expression of LAP3 exhibited a significant increase in the 6-h group, and this increase was further enhanced in the 12-h and 24-h groups when compared to the 0-h group.

MAGE family member D1 (MAGED1) refers to an adaptor for ubiquitin-dependent degradation pathways and a transcriptional co-factor that plays a vital function in cell differentiation and circadian rhythm. MAGED1 represents a new regulator of osteoblastogenesis, osteoclastogenesis, and bone remodeling in a mouse model. MAGED1 is discovered to be a novel negative regulator of PPARγ activity, adipogenesis, as well as insulin sensitivity in mice (54). We observed that the mRNA expression of MAGED1 was significantly lower in the 12-h-induced group and even lower in the 24-h-induced group when compared to the 0-h group.

Epiphycan (EPYC), also known as dermatan sulfate proteoglycan 3 (DSPG3), is a member of the small leucine-rich proteoglycan family and is associated with extracellular matrix organization (55). Recent studies have suggested that EPYC is involved in extracellular matrix remodeling and IL-17-related signaling in joint tissues, indicating a potential role in disease-associated changes in cartilage homeostasis (56). In addition, EPYC has been identified as a feature gene in osteoarthritis and may be associated with immune infiltration, further supporting its relevance in joint disease biology (57). EPYC has also been implicated in non-articular tissues, including the cornea, although the underlying mechanisms remain incompletely understood (58). Previous bioinformatics studies have identified EPYC as an osteoarthritis-related feature gene, and its expression has been reported to be significantly increased in osteoarthritis compared with healthy controls (59). In addition, ROC analysis showed that the AUC values of the three characteristic genes, EPYC, MAGED1, and LAP3, were all above 0.85 in the dataset. Our findings suggest EPYC, MAGED1, and LAP3 as potential diagnostic biomarkers for RA. Future studies including healthy control cohorts may further evaluate the broader diagnostic spectrum of these biomarkers and better assess their diagnostic performance across different clinical contexts. We further analyzed these genes by LASSO and SVM-REF methods and found that EPYC and LAP3 are related to most immune cells in RA, while LAP3 is related to most immune cells in osteoarthritis. Conversely, the mRNA expression of EPYC exhibited a significant increase in the 6-h, 12-h, and 24-h-induced groups when compared to the 0-h group. Concerning EPYC, there was no significant difference in protein expression between the 6-h and 12-h-induced groups when compared to the 0-h group; however, a significant increase was observed in the 24-h-induced group.

By searching for transcription factors that could regulate the characteristic genes through the Enrichr online tool, MEF2A and YY1 were found to have potential regulatory effects. MEF2A is a vital transcriptional regulatory factor essential for cell differentiation, cell proliferation, cell survival, and morphogenesis (60, 61). YY1 plays a key role in stimulating IL-6 transcription in RA, contributing to inflammation in RA through the stimulation of Th17 differentiation (62). YY1 also participates in RA neutrophil infiltration through the PI3K/Akt/mTOR/IL-8 signal pathway (63). Our analysis showed that the transcription factors MEF2A and YY1 might regulate the characteristic genes; however, this finding requires further experimental validation.

Drug sensitivity analysis was performed based on CellMiner cancer cell line IC50 data to explore potential compounds associated with the expression levels of the identified genes. It should be noted that this analysis was exploratory. The identified compounds, including tegafur, fenretinide, and alectinib, were statistically correlated with the characteristic genes in cancer cell models. However, these findings do not indicate therapeutic efficacy in RA and may only provide preliminary clues for future drug repositioning studies. Further validation in RA-specific cellular or animal models is still needed to determine their potential relevance.

In this study, we used SVM-RFE and LASSO logistic regression to identify diagnostic markers of RA and to explore immune cell infiltration in RA tissues. Nevertheless, this study still has several limitations. First, the sample size was relatively small. Therefore, studies with larger sample sizes and prospective cohorts are needed to further verify our findings.

## 5 Limitations

Several limitations of this study should be acknowledged. First, the overall sample size of the included transcriptomic datasets was relatively limited, which may increase the risk of model overfitting and reduce statistical power. Although cross-validation was applied, independent external validation using larger multicenter cohorts is necessary to confirm generalizability. Given the limited sample size, threshold-dependent metrics such as sensitivity and specificity may be unstable and cutoff dependent; therefore, we used the AUC as the primary, threshold-independent measure of discrimination in this study. Second, the study design was cross-sectional and based on public transcriptomic data; therefore, causal relationships between the identified genes and RA pathogenesis cannot be established. Longitudinal cohort studies or functional gain- and loss-of-function experiments are needed to clarify potential causal roles. Third, drug sensitivity analysis was performed using pharmacological data derived from cancer cell lines, which may not directly reflect drug responses in RA synovial fibroblasts. Fourth, experimental validation was limited to mRNA expression in a single RA fibroblast-like synoviocyte cell line. Protein-level validation and functional mechanistic studies are needed to strengthen the biological interpretation of our findings further. Finally, the PPI network analysis revealed relatively weak connectivity; therefore, these results should be interpreted as exploratory rather than as definitive mechanistic evidence.

In summary, EPYC, MAGED1, and LAP3 were identified as inflammation-associated genes with potential diagnostic relevance in distinguishing RA from OA. These findings provide preliminary molecular insights into RA pathogenesis. Further validation in independent clinical cohorts and mechanistic studies are needed before clinical application.

## Data Availability

The original contributions presented in the study are included in the article/Supplementary material, further inquiries can be directed to the corresponding author.

## Footnotes

1
http://r-project.org/

2
https://www.ncbi.nlm.nih.gov/geo/

3
https://www.genecards.org/

4
https://string-db.org/

5
http://mirdb.org/

6
https://mirtarbase.cuhk.edu.cn/

7
https://maayanlab.cloud/Enrichr/
